# Combatting antibiotic resistance in *Gardnerella vaginalis*: A comparative *in silico* investigation for drug target identification

**DOI:** 10.1371/journal.pone.0314465

**Published:** 2025-03-12

**Authors:** Rabbia Riaz, Kanwal Khan, Saltanat Aghayeva, Reaz Uddin

**Affiliations:** 1 Baqai Institute of Information Technology, Baqai Medical University, Karachi, Pakistan; 2 Dr. Panjwani Center for Molecular Medicine and Drug Research, International Center for Chemical and Biological Sciences, University of Karachi, Karachi, Pakistan; 3 Western Caspian University Baku, Baku, Azerbaijan; Universidade dos Açores Departamento de Biologia: Universidade dos Acores Departamento de Biologia, PORTUGAL

## Abstract

*Gardnerella vaginalis* is the most frequently identified bacterium in approximately 95% of bacterial vaginosis (BV) cases. This species often exhibits resistance to multiple antibiotics, posing challenges for treatment. Therefore, there is an urgent need to develop and explore alternative therapeutic strategies for managing bacterial vaginosis. The objective of this study was to identify virulence factors and potential drug targets against *Gardnerella vaginalis* by utilizing *in silico* methods, including subtractive and comparative genomics. These methods enabled the systematic comparison of genetic sequences to pinpoint specific features unique to *G. vaginalis* and crucial for its pathogenicity, which could then inform the development of targeted therapeutic strategies. The analysis of the pathogen's proteomic data aimed to identify proteins that fulfilled specific criteria. These included being non-homologous to human proteins, essential for bacterial survival, amenable to drug targeting, involved in virulence, and contributing to antibiotic resistance. Following these analyses and an extensive literature review, the phospho-2-dehydro-3-deoxyheptonate aldolase enzyme emerged as a promising drug target. To deepen our understanding of the biological function of the identified protein, comprehensive protein structural modeling, validation studies, and network topology analyses were conducted. The subsequent structural analysis, encompassing modeling, validation, and network topology assessment, is aimed at further characterizing the protein. Using a library of around 9,000 FDA-approved compounds from the DrugBank database, a virtual screening was conducted to identify potential compounds that could effectively target the proposed drug target. This approach facilitated the evaluation of existing drugs for their ability to inhibit the target, potentially offering an efficient pathway for developing new treatments against the pathogen. Leveraging the established efficacy, safety, pharmacokinetics, and pharmacodynamics of these compounds, the study suggests repurposing them for *Gardnerella vaginalis* infections. Among the screened compounds, five specific agents—DB03332, DB07452, DB01262, DB02076, and DB00727—were identified as cost-effective therapeutic options for treating infections related to *Gardnerella vaginalis*. These compounds were selected based on their efficacy in targeting the pathogen while maintaining economic feasibility. While the results indicate potential efficacy in treating infections caused by the pathogen, further experimental studies are essential to validate these findings.

## 1. Introduction

Bacterial vaginosis (BV), a dysbiosis of the vaginal microbiota, has been associated with several detrimental health outcomes. The condition is characterized by low concentrations of “healthy” lactobacilli and an excess of various bacteria from other taxonomic groups. BV has been linked to an increased risk of STIs, UTIs, post-surgical complications, infertility, and pregnancy losses. Eukaryotic infections, such as trichomoniasis, as well as viral infections caused by HIV, HSV, and HPV, can also contribute to the development of these diseases and the health conditions associated with BV [1]. In addition, Women with BV-negative microbiomes are more prone to experiencing genital irritation and vaginal colonization by other possible pathogens, such as beta-hemolytic streptococci and *Fusobacterium nucleatum*. *Gardnerella vaginalis* lacks the classical cell-wall lipopolysaccharide and has a gram-positive cell-wall ultrastructure [2].

Women who had bacterial vaginosis were exposed to strains of *Gardnerella vaginalis* that were resistant to metronidazole, clindamycin, and amoxicillin/clavulanic acid. The most common and well researched pathogen associated with BV, *G. vaginalis* has been isolated in approximately 95% of cases [2]; the prevalence varies depending on race and ethnicity. According to the National Health and Nutrition Examination Survey 2001–2004, 29.2% of American women of reproductive age had BV, yet only 15.7% of these women reported having vaginal symptoms [3]. Furthermore, a study conducted by Zhang *et al*, involving 1,218 married Chinese women, revealed that bacterial vaginosis (BV) ranked as the second most prevalent disorder, with an estimated frequency of 10% [4]. Additionally, a separate investigation by Dai *et al* reported a notably higher prevalence of BV, reaching 51.6%, in the Tibetan region of Sichuan Province, China [5]. BV can be categorized as a biofilm-associated infection because *G. vaginalis* is dominant in the densely organized polymicrobial biofilm that adheres to the vaginal epithelium [6]. The Centers for Disease Control and Prevention (CDC) advises using oral or vaginally administered metronidazole or clindamycin as the first-line of treatment for BV [7]. Widely employed is the nitro imidazole derivative metronidazole. It can be applied intravaginally once daily for five days in the form of a 0.75% gel, or given orally twice daily for seven days at a dose of 500 mg [8].

Clindamycin (Cleocin), metronidazole (Flagyl), and metronidazole vaginal gel (Metro Gel-Vaginal) are all examples of antibiotics used for the treatment of BV. Ciprofloxacin, cefuroxime, ceftazidime, as well as several other antibiotics traditionally used for the treatment of BV include metronidazole, erythromycin, chloramphenicol, ceftriaxone, and cloxacillin. *G. vaginalis* isolates have developed resistance to Penicillin, ampicillin, tetracycline, and gentamycin whereas the pathogen displayed moderate resistance to streptomycin and Augmentin. Traditional approaches using experimental techniques are time consuming and laborious. Computational approaches such as subtractive genomics are a preferable alternative, which give us an insight into the emergence of pathogenic bacteria and allows interpretation of transmission events. Subtractive genomics is a widely utilized technique which is used to identify a group of proteins that are unique and necessary for the bacteria’s survival but are not present in the host. These proteins were retained for analysis using bash scripting. Bacterial vaginosis (BV) and trichomoniasis, which account for 60–80% of vaginitis cases are the most frequent causes of the condition [9]. Although there are several pharmacological compounds available to treat this disorder, recurrence and the emergence of drug resistance prevent the efficacy of current therapeutic interventions. Additionally, affecting women's fertility, recurrent vaginitis raises the chance of preterm birth, causes secondary infections, and lowers quality of life [9,10]. Studies have also suggested a link between a woman's education and socioeconomic status and her likelihood of contracting the virus [11,12].

The objective of the current study is to identify an effective therapeutic target for treating infections caused by *Gardenerella vaginalis via* the use of a subtractive genomics methodology. We have applied molecular docking and virtual screening approaches to shortlist efficacious therapeutic agents against the pathogen. Lastly, the ADMET profiling of these shortlisted therapeutic agents was performed.

## 2. Materials and methods

**Fig 1:**
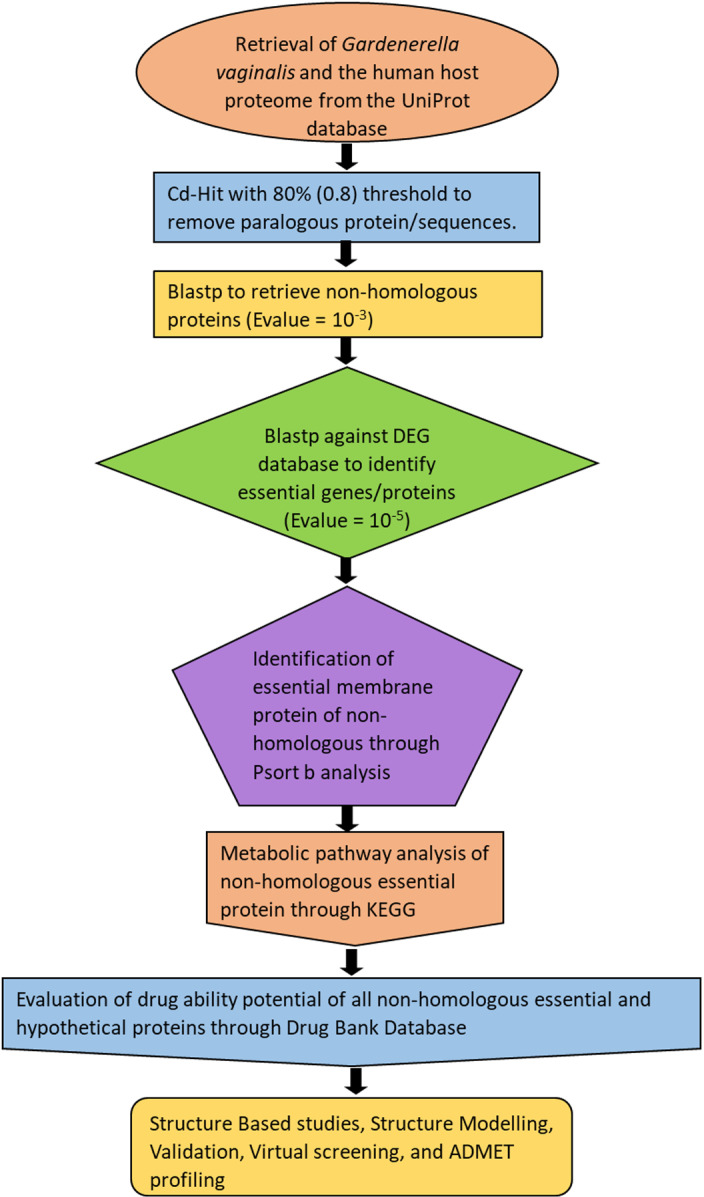
Flowchart of Research Study. A brief overview of the research methodology.

The subtractive genomics approach was performed to identify a potential drug target. The methodology of the study is shown in the flowchart below Fig 1:

### 2.1 Data retrieval

Universal Protein Resource (UniProt) Database was used for the retrieval of proteome sequences [13]. The FASTA sequence of *Gardenerella vaginalis* (strain 6420B; UP000033124) contained the full proteome. The sequencing of *Gardenerella vaginalis* contained 1142 distinct protein counts. The same database was used for the retrieval of the Human proteome (UP000005640) in FASTA sequence format. As per the UniProt annotation, the Human proteome consists of a protein count of 82,492. Among this protein count of the proteome sequence, TrEMBL sequences as well as the species’ identified sequences both were included. Additionally, the Kyoto Encyclopedia of Genes and Genomes (KEGG) database was utilized for the prediction of metabolic pathways [14]. The DrugBank database was used for the screening of druggable proteins [15]. The study also involves the use of the Virulence Factor Database (VFDB) to check the virulence factors of the pathogen’s essential proteins [16]. Lastly, to assess the essentiality of the drug targets the Database of Essential Genes (DEG) was used [17].

### 2.2 Finding paralogous sequences

The CD-HIT database's clustering techniques were used to find the non-paralogous protein sequences [18]. A score of 0.8 was chosen as the cutoff point for eliminating redundant *Gardenerella vaginalis* sequences. Protein sequences that shared more than 80% of their similarities were eliminated as paralogous sequences. For additional investigation, only the non-paralogous were retained for further analysis [19].

### 2.3 Identification of non-homologous proteins

In order to find the non-homologous proteins in the pathogen, a BLASTp was implemented against the proteome of *H. sapiens* using the protein sequences that we obtained from the CD-HIT results. The E-value for BLASTp was set to 10^-3^. The BLASTp gave us “Hits,” which are sequences that are similar to or identical to the pathogen and host, and “No Hits,” which are sequences that are neither similar to nor identical to the pathogen and host. The “No Hits” sequences were thus retained for further analysis because the primary goal of BLAST was to find sequences non-homologous to those found in the human proteome. The selection provided us with protein sequences that are unique to the pathogen and may be used for further investigation without sharing any functional similarities with human proteins, ensuring that there would be the least amount of cross-reactivity possible [20].

### 2.4 Identification of essential non-homologous proteins

The most recent edition of the Database of Essential Genes (DEG) is accessible at http://tubic.tju.edu.cn/deg/, where annotations of essential genes can be downloaded. The DEG database contains 39 bacterial essential gene sets. Proteins that are crucial for cellular metabolism or those needed for an organism's growth and survival are known to be crucial for the pathogen’s survival [20]. One of the crucial first steps is the finding of these crucial proteins because it can act as a checkpoint for target choice and crucial microbial cellular function [21]. With the E-value set at 10^-5^, BLASTp was used to compare the previously discovered non-homologous protein to the DEG database and screen for protein sequences critical to the pathogen's survival. The ‘Hits’ from this BLAST were used to move on to the next step, which involved looking up these essential proteins found in the pathogen against the DrugBank database [22].

### 2.5 Druggability of essential genes

We used an E-value of 10^-3^ to compare the essential non-homologous proteins we received from the DEG database for comparison against the DrugBank database. The goal of this screening was to identify the proteins that potentially serve as therapeutic targets. Protein sequences that had a high degree of similarity to the database in the BLAST results were considered as potential drug targets.

### 2.6 Identification of virulent proteins

The proteins that cause infection in the host are known as virulent proteins, and they are vital for both the pathogenesis and virulence of the organism. In addition to aiding the pathogen in infecting the host, these virulent factors also assist in weakening the host's immune system through invasion, adhesion, and colonization, which leads to the emergence of a diseased condition. Using BLASTp with a cutoff E-value of 10^-5^, the outcomes from the preceding phase were compared to the Virulence Factor Database (VFDB). The sequences retrieving ‘Hits’ against the VFDB database were retrieved for further analysis [23].

### 2.7 Screening of resistance proteins

The process of treating infectious diseases has become more complex due to the rise in drug-resistant infections and the loss of potency of many medications. In light of the previous finding, BLASTp was used to search the ARG-ANNOT (Antibiotic Resistance Gene-Annotation) database [24] for antibiotic resistant proteins. Such protein sequences that have been experimentally shown to be resistant to a variety of antibiotic classes, such as lactamases, fluoroquinolones, aminoglycosides, Fosfomycin, trimethoprim, and sulfonamide, are available in the ARG-ANNOT database [24]. An E-value of 10^-5^ was used for the BLAST run. The sequences retrieving ‘Hits’ against the database were retained for further analysis while the ‘No Hits’ results were discarded [25].

### 2.8 Prediction of sub-cellular localization

Finding an appropriate therapeutic target and genome functional annotation depends on the potential drug target’s sub-cellular localization. The sub-cellular localization of the proteins was assessed using the PSORTb v.3.0 and the CELLO tool v.2.5 [26,27] to assess the retained drug target’s biological and functional location. With the aid of these techniques, the sub-cellular localization of proteins was predicted.

### 2.9 Host and pathogen metabolic pathway analysis

The protein that was selected using this subtractive genomics method underwent additional analysis utilizing the KEGG database [28]. To identify common and unique metabolic pathways, a comparison between the host and the pathogen was also performed. In contrast to common pathways, which are found in both the host and the pathogen, unique pathways are those that are only present in the pathogen.

### 2.10 Structure modelling of the selected protein

Downstream analysis of the protein required that the 3D structure of the protein be known. Since the potential drug target’s 3D was not available online the stand alone tool MODELLER had to be utilized [22]. The MODELLER program simulates the three-dimensional (3D) structures of proteins as well as their assembly under spatial constraints. MODELLER is mostly used for homology modelling or comparative protein structural modelling [29]. The generated 3D structure was subsequently validated through stereochemical analysis using PROCHECK [30] and 3D structure modelling validation was performed using ProSA web servers [31].

### 2.11 Molecular docking and virtual screening

The protein structure obtained was subjected to docking using AutoDock version 4.2 [32]. Through AutoDock, we examined the interactions within the protein-ligand complex. The complex displaying the most favorable binding energy from the initial docking was chosen as a reference for subsequent re-docking of the protein and ligand. Among the 250 conformations generated during re-docking, the complex with the most favorable binding energy was selected for further analysis. Furthermore, Discovery Studio and UCSF Chimera v.1.14 were employed to generate and analyze the three-dimensional interactions of the re-docked complex [33].

The protein was then virtually screened against the FDA-approved library of the DrugBank database. The library used for screening comprised a total of 9,213 drugs. These medications were virtually screened against the potential drug target. The primary goal of the virtual screening process was to propose the re-purposing of these FDA approved drugs as therapeutic agents to combat infections caused by the pathogen. After screening, the substances with the most favorable binding energies were chosen for ADMET profiling to ascertain their pharmacodynamic and pharmacokinetic properties.

### 2.12 ADMET profiling

A chemical compound's ADMET properties include parameters such as absorption, metabolism, distribution, excretion by the human body as well as its toxicity to the human body [20]. SwissADME, an online tool, can be used to evaluate the medicinal chemistry, drug-likeness, and pharmacokinetics of the shortlisted compounds [34]. Additionally, pkCSM was used for the screening of the shortlisted therapeutic agents’ toxicity characteristics [35].

### 2.13 Protein–protein interaction

Cellular systems are composed of numerous interactomes. In order to find out how the shortlisted proteins interact with other proteins, the STRING (Search Tool for the Retrieval of Interacting Genes/Proteins) database was utilized [36]. There are many different 3D-predicted, and empirically verified PPIs available within the database.

## 3. Results

### 3.1 Identification of non-paralogous sequences

The complete proteome of *Gardenerella vaginalis* (strain 6420B) consisting of 1142 protein was retrieved from the UniProt database. From a total of 1142 proteins, CD-HIT determined that 1139 were unique and non-paralogous. Since paralogous sequences are considered to be redundant proteins, these proteins are discarded from further analysis [37]. This step helped to identify 1139 non-paralogous protein sequences which were then retained for further analysis.

### 3.2 Identification of the non-homologous sequences

To find the non-homologous sequences, BLASTp implemented using the remaining non- paralogous 1139 protein sequences as a query against the human proteome. Out of the 1139 proteins, 733 were found to be non-homologous, i.e., unique to the pathogen. The homologous sequences were excluded to prevent host cytotoxicity caused by therapeutic agents that would be proposed in the study.

### 3.3 Identification of essential non–homologous genes

The 733 non- homologous *Gardenerella vaginalis* were subjected to a BLASTp search against the DEG database. The BLASTp result provided 376 proteins that were found to be essential as they were involved in such activities that are significant to pathogen’s biological pathways. We can therefore argue that these critical non-homologous proteins can serve as prospective therapeutic targets against the pathogen. Targeting such proteins threatens the survival of the pathogen.

### 3.4 Druggability of the essential genes

The resultant non-homologous essential genes were then used as a query when the BLASTp was implemented against the DrugBank database. The BLASTp was performed to find out the sequences that are druggable among the 376 non-homologous essential proteins. Only the protein sequences which showed similarity to the FDA-approved drug targets were used for further downstream analysis. As a result, 118 out of 376 were found to be non-homologous, essential, and viable therapeutic targets after retrieving “HITS” against the database. The remaining 258 protein sequences, however, were discarded.

### 3.5 Identification of the virulent protein

One of the most important elements which ensure the survival of bacteria is virulence. Virulent proteins help pathogens evade the host’s immune system and thereby ensure the pathogen’s survival. Therefore, BLASTp was implemented using the shortlisted 118 proteins as a query against the Virulent Factor Database. As a result, 34 proteins were retained based on their significant similarity to virulent proteins present within the database.

### 3.6 Screening of antimicrobial resistant proteins

The emergence of bacterial resistance is heavily influenced by proteins engaged in antibiotic resistance mechanisms. Consequently, these proteins offer a potential avenue for the development of therapeutic agents to address infections attributed to *Gardenerella vaginalis* (strain 6420B). A total of 10 proteins have been identified as crucial components in the metabolism of antibiotics utilized for treating infections caused by this pathogen. These proteins were selected following a BLASTp analysis against the ARG-ANNOT database. Targeting these proteins presents an opportunity to diminish the pathogen's resistance, as it may impede crucial bacterial pathogenesis pathways. Table 1 provides an overview of the proteins retained at each stage of the filtering process, illustrating the number of proteins retrieved after applying each filtering parameter.

**Table 1:** Subtractive filtering of proteins from *Gardenerella vaginalis* in the current investigation.

S. No.	Steps involved in the current study	Number of proteins
1	Complete proteome of *Gardenerella vaginalis*	1142
2	Number of non-paralogous sequences (after CD-HIT)	1139
3	BLASTp of proteome against human host proteome (E-value 10 ^− 3^)	733
4	BLASTp of non-homologous proteome against DEG (E-value 10 ^− 5^)	376
5	BLASTp of non-homologous essential proteome against DBD (E-value 10 ^− 3^)	118
6	BLASTp of non-homologous essential proteome against VFDB (E-value 10 ^− 5^)	34
7	BLASTp of non-homologous essential proteome against ARG-ANNOT (E-value 10 ^− 5^)	10

### 3.7 Prediction of sub-cellular localization

The proteins selected in step 6, as outlined in Table 1, underwent sub-cellular location prediction to identify the most promising pharmacological target. Although the filtering process ultimately identified 10 key proteins related to antibiotic metabolism, an additional analysis of 34 proteins was performed to explore a broader subset for potential targets. This step aimed to ensure that all relevant proteins were considered for their therapeutic potential.

**Fig 2:**
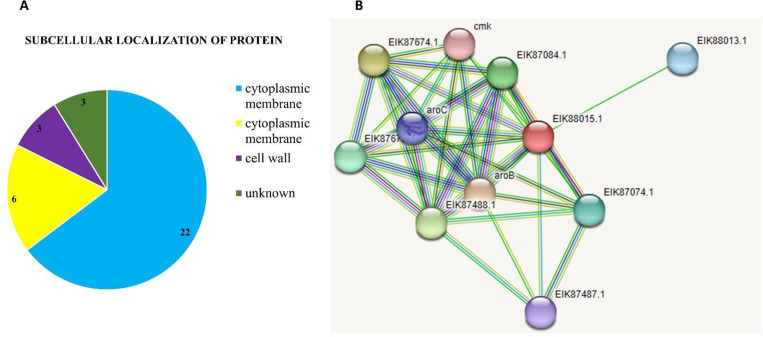
(A) A diagram displaying the shortlisted proteins’ subcellular localization (B) A protein-protein interaction diagram utilizing the STRING database.

Out of the 34 proteins analyzed, 22 were determined to be cytoplasmic, 6 were situated in the cytoplasmic membrane, 3 remained unidentified in terms of sub-cellular location, and 3 were located in the cell wall. The sub-cellular localization, conducted using PSORTb, facilitated the precise identification of our proteins of interest, allowing for a focused consideration. From this subset, three proteins—pullalanase, putative xylan esterase, and the cytoplasmic protein phospho-2-dehydro-3-deoxyheptonate aldolase—were chosen for further evaluation as potential therapeutic targets Fig 2.

#### 3.7.1 Significance of proposed drug target.

Many organisms, including bacteria, plants, and fungi use the enzyme phospho-2-dehydro-3-deoxyheptonate aldolase (PDA) to create aromatic amino acids including phenylalanine, tyrosine, and tryptophan. It causes the condensation of 3-deoxy-D-arabino-heptulosonate 7-phosphate (DAHP) and erythrose 4-phosphate (E4P) to produce phosphoenolpyruvate (PEP). This reaction is precursor to the shikimate pathway, which produces aromatic chemicals.

In addition to aromatic amino acids, the shikimate pathway also generates many secondary metabolites, including phenolic compounds, flavonoids, lignin, and alkaloids, which are precursors for the production of a wide range of important chemicals. These substances are involved in numerous biological processes including signal transduction, antioxidant activity, protein synthesis, and serve as defense mechanisms against herbivores and pathogens. Due to the actions of this protein, *Gardenerella vaginalis* can be treated using this protein as a drug target [38].

### 3.8 Protein-protein interactions

The protein Phospho-2-dehydro-3-deoxyheptonate aldolase was searched in the STRING database, and it was discovered that this protein interacts with various other functional proteins (Table 2).

**Table 2:** List of STRING interactions shown in tabular format.

NODE 1	NODE 2	ANNOTATIONS	FAMILY	SCORE
EIK88015.1	EIK87074.1	Phospho-2-dehydro-3-deoxyheptonate aldolase; Stereospecific condensation of phosphoenolpyruvate (PEP) and D-erythrose-4-phosphate (E4P) giving rise to 3-deoxy-D-arabino- heptulosonate-7-phosphate (DAHP)	Belongs to the transketolase family	0.723
EIK88015.1	EIK87084.1	Phospho-2-dehydro-3-deoxyheptonate aldolase; Stereospecific condensation of phosphoenolpyruvate (PEP) and D-erythrose-4-phosphate (E4P) giving rise to 3-deoxy-D-arabino- heptulosonate-7-phosphate (DAHP)	COG0169 Shikimate 5-dehydrogenase	0.695
EIK88015.1	EIK87487.1	Phospho-2-dehydro-3-deoxyheptonate aldolase; Stereospecific condensation of phosphoenolpyruvate (PEP) and D-erythrose-4-phosphate (E4P) giving rise to 3-deoxy-D-arabino- heptulosonate-7-phosphate (DAHP)	Fructose-bisphosphate aldolase; Catalyzes the aldol condensation of dihydroxyacetone phosphate (DHAP or glycerone-phosphate) with glyceraldehyde 3-phosphate (G3P) to form fructose 1,6-bisphosphate (FBP) in gluconeogenesis and the reverse reaction in glycolysis	0.695
EIK88015.1	EIK87488.1	Phospho-2-dehydro-3-deoxyheptonate aldolase; Stereospecific condensation of phosphoenolpyruvate (PEP) and D-erythrose-4-phosphate (E4P) giving rise to 3-deoxy-D-arabino- heptulosonate-7-phosphate (DAHP)	COG1605 Chorismate mutase	0.868
EIK88015.1	EIK87674.1	Phospho-2-dehydro-3-deoxyheptonate aldolase; Stereospecific condensation of phosphoenolpyruvate (PEP) and D-erythrose-4-phosphate (E4P) giving rise to 3-deoxy-D-arabino- heptulosonate-7-phosphate (DAHP)	Chorismate mutase; COG0077 Prephenate dehydratase	0.887
EIK88015.1	EIK87675.1	Phospho-2-dehydro-3-deoxyheptonate aldolase; Stereospecific condensation of phosphoenolpyruvate (PEP) and D-erythrose-4-phosphate (E4P) giving rise to 3-deoxy-D-arabino- heptulosonate-7-phosphate (DAHP)	COG0287 Prephenate dehydrogenase	0.743
EIK88015.1	EIK88013.1	Phospho-2-dehydro-3-deoxyheptonate aldolase; Stereospecific condensation of phosphoenolpyruvate (PEP) and D-erythrose-4-phosphate (E4P) giving rise to 3-deoxy-D-arabino- heptulosonate-7-phosphate (DAHP)	5-methylthioadenosine nucleosidase; Catalyzes the irreversible cleavage of the glycosidic bond in both 5’-methylthioadenosine (MTA) and S-adenosylhomocysteine (SAH/AdoHcy) to adenine and the corresponding thioribose, 5’- methylthioribose and S-ribosylhomocysteine, respectively	0.721
EIK88015.1	aroB	Phospho-2-dehydro-3-deoxyheptonate aldolase; Stereospecific condensation of phosphoenolpyruvate (PEP) and D-erythrose-4-phosphate (E4P) giving rise to 3-deoxy-D-arabino- heptulosonate-7-phosphate (DAHP)	Bifunctional shikimate kinase/3-dehydroquinate synthase; Catalyzes the conversion of 3-deoxy-D-arabino-heptulosonate 7-phosphate (DAHP) to dehydroquinate (DHQ)	0.998
EIK88015.1	aroC	Phospho-2-dehydro-3-deoxyheptonate aldolase; Stereospecific condensation of phosphoenolpyruvate (PEP) and D-erythrose-4-phosphate (E4P) giving rise to 3-deoxy-D-arabino- heptulosonate-7-phosphate (DAHP)	Chorismate synthase; Catalyzes the anti-1,4-elimination of the C-3 phosphate and the C-6 proR hydrogen from 5-enolpyruvylshikimate-3-phosphate (EPSP) to yield chorismate, which is the branch point compound that serves as the starting substrate for the three terminal pathways of aromatic amino acid biosynthesis. This reaction introduces a second double bond into the aromatic ring system	0.712
EIK88015.1	cmk	Phospho-2-dehydro-3-deoxyheptonate aldolase; Stereospecific condensation of phosphoenolpyruvate (PEP) and D-erythrose-4-phosphate (E4P) giving rise to 3-deoxy-D-arabino- heptulosonate-7-phosphate (DAHP)	Bifunctional cytidylate kinase/gtp-binding protein; GTPase that plays an essential role in the late steps of ribosome biogenesis	0.629

The protein is involved in the stereospecific condensation of phosphoenolpyruvate (PEP) and D-erythrose-4-phosphate (E4P) giving rise to 3-deoxy-D-arabino-heptulosonate-7-phosphate (DAHP). The phosphor-2-dehydro-3-deoxyheptonate aldolase was represented by a network consisting of ten nodes.

### 3.9 Protein homology modelling and structural validation

**Fig 3:**
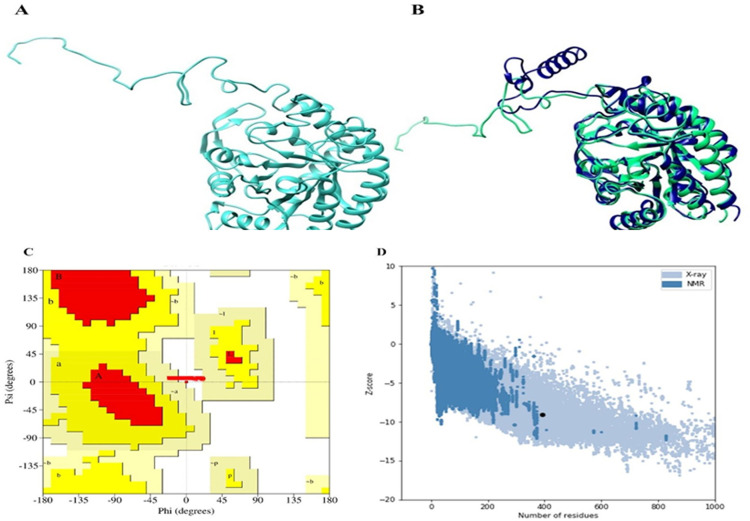
(A) modeled structure of phospho-2-dehydro-3-deoxyheptonate aldolase and (B) shows the 4HSO Template protein (green color) superimposed on the modeled protein structure (blue color) Having the percent identity of 88% (C) the PROCHEK validation of modeled structure, and (D) The Prosa web score (z = -9.11) for the phospho-2-dehydro-3-deoxyheptonate aldolase structure.

Since the protein's PDB structure was not available, the protein’s FASTA sequence available in the UniProt database under the accession ID I4LQB8 was utilized to model the protein’s three-dimensional structure. PDB ID 4HSO (shown in Fig 3A), and the I4LQ8B superimposed structure have been displayed.

The Ramachandran Plot was used to validate the protein’s modelled 3D structure. According to the ProCheck server's findings, out of the protein’s 393 residues, 96.2% were in the most favorable region (Fig 3C). The generated 3D structure was also validated using the ProSA web server. A negative Z-score denoted an error-free structural model. Since the modelled protein had a Z-score of -9.11 it was retained for further analysis (Fig 3D).

### 3.10 Molecular docking of the protein

**Fig 4:**
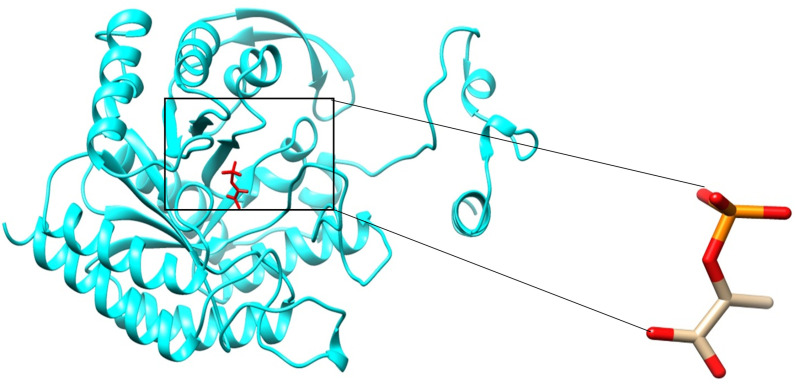
Redocking of a co-crystallized phospho-2-dehydro-3-deoxyheptonate aldolase ligand (cysteine) within the phospho-2-dehydro-3-deoxyheptonate aldolase active site, displaying an RMSD of 1.9Å.

Using Auto Dock v4.2, the phospho-2-dehydro-3-deoxyheptonate aldolase docking analysis was completed. Lamarckian Genetic Algorithm was used with the population size set to 300 times with a maximum of 2,500,000 evaluation steps, resulting in 27,000 generations. Ten runs were implemented for the docking of the ligand to the protein. The Auto Dock outcomes showed that the ligand interacted with the protein's active site in a variety of conformations and orientations. The central grid parameters were 8, 12, and 16 for the X, Y and Z coordinates, respectively. Re-docking was performed using the conformation of the best docked protein-ligand complex as a reference. As a result, the Root-Mean-Square Deviation (RMSD) was found to be 1.931Å (Fig. 4). The best docked binding energy is -4.827 kcal/mol. This outcome is consistent with ligand and active site spontaneous binding which results in the formation of a lower energy complex that is more stable. The protein was then virtually screened against a library of FDA-approved medicinal compounds.

### 3.11 Virtual screening

**Fig 5:**
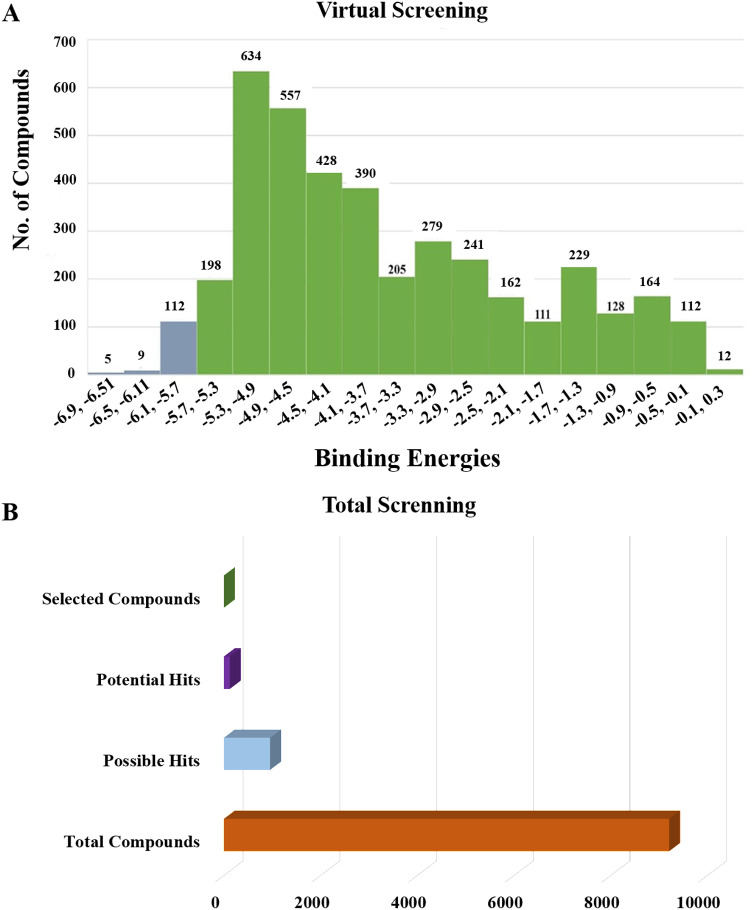
Virtual screening results of the 9213 FDA approved drugs against phospho-2-dehydro-3-deoxyheptonate aldolase (A) The virtual screening results of the protein against the FDA Approved library showed that the binding affinities ranged from -6.9 to 0.3 kcal/mol. (B) selecting a small number of these chemicals to be used in the current investigation.

Following the molecular docking of the selected protein and its ligand, the optimal docking conformation was virtually screened against a library of more than 9213 medicinal compounds that have been approved by the FDA. Finally, the binding energies ranged from -6.9 kcal/mol to 0.3 kcal/mol after the virtual screening of the ~ 9000 compounds against phospho-2-dehydro-3-deoxyheptonate aldolase were performed (Fig 5A). The bar graph shows the virtual screening of 4064 compounds among which five compounds have been selected that have binding energies ranging from -5.9 to -6.9 kcal/mol. These compounds can be categorized as potential hits if they had binding energies comparable to the reference ligand (-4.827 kcal/mol). Thus, as seen in Fig 5B, 126 molecules had high binding energies ranging from -5.9 to -6.9 kcal/mol. In the current study, 5 compounds were selected as best lead compounds against phospho-2-dehydro-3-deoxyheptonate aldolase protein with binding energies from -6.9 to -5.9 to kcal/mol suggesting the strongest inhibition, i.e., (i) DB03332 (5,6-Cyclic-Tetrahydropteridine). This is an experimental small molecule. Information as to what types of diseases this drug treats was not available. (ii) DB07452 (2,6-diamino-1,7-dihydro-8H-imidazo[4,5-g] quinazolin-8-one) is also an experimental small molecule. Information as to what types of diseases this drug treats was not available. (iii) DB01262 (Decitabineand) is used for the treatment of myelodysplastic syndromes (MDS) by inducing DNA hypomethylation and corresponding alterations in gene expression. (iv) DB02076 (6-phospho-D-gluconic acid) is an experimental small molecule. Information as to what types of diseases this drug treats was not available. (v)DB00727 (Nitroglycerin) is a nitrate vasodilator used to treat or prevent angina, heart failure, hypertension, and anal fissures. These 5 compounds having binding energies -6.9.-6.6,-6.5,-6.0 and -5.9 kcal/mol, respectively. Fig 5 shows the virtual screening results of the 9213 FDA approved drugs against phospho-2-dehydro-3-deoxyheptonate aldolase.

### 3.12 Interaction analysis of shortlisted compounds

To better understand the binding mechanism that existed within the protein-drug complex, a post-molecular docking interaction was performed on the shortlisted drugs. The green color shows conventional hydrogen bond and the light orange color shows pi-cation and pi-anion bond and light pinkish color shows pi-pi stacked and the light purple color shows pi-alkyl bond.

**Fig 6:**
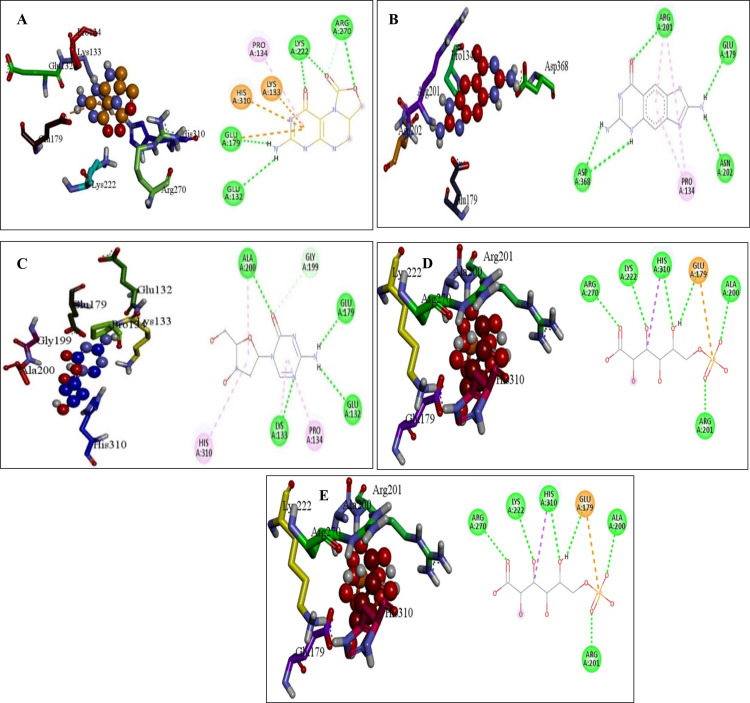
A molecular docking analysis of the molecules DB03332, DB07452, DB01262, DB02076 and DB00727 demonstrating the 2D interaction of ligands produced using Discovery studio shown as (A), (B), (C), (D) and (E), respectively.

According to the docking study, DB03332 ultimately demonstrated the most favorable binding energy among the selected molecules. The compound formed seven hydrogen bonds, three electrostatic contacts, and hydrophobic interactions with His310, Lys133, and Pro134 at a distance of 5.05, 5.34 and 4.37 Å. The interaction between residue and the compound had a net energy of -6.9 kcal/mol (Fig 6A). DB07452 formed a complex with the protein with a binding energy of -6.6 kcal/mol. It formed five hydrogen bonds, and four hydrophobic bridges between the amino acids ARG201, ASP368, ASP368, GLU179, ASN202, PRO134, ARG201, PRO134 and ARG201 (Fig 6B). Additionally, the DB01262 molecule interacted with Lys133, ALA200, GLU179, GLU132, GLY199, ALA200, HIS310, LYS133 and PRO134 via four hydrophobic, and six hydrogen bonds resulting in a binding energy of -6.5 kcal/mol (Fig 6C). Moreover, the DB02076 formed eight hydrogen bonds, one hydrophobic and one electrostatic bond with GLU179, ALA200, ARG201, LYS222, ARG270, HIS310, GLU179, ARG270 and HIS310 with bond lengths of 2 in hydrogen bonding and 3 in electrostatic and hydrophobic bonds. The complex had a binding affinity of -6.0 kcal/mol (Fig 6D). Lastly, the DB00727 formed a complex with the protein with a binding energy of -5.9 kcal/mol. The compound interacted with ARG128, LYS133, LYS133, LYS133, LYS222, LYS222, HIS310, ARG128, ARG128, ARG128, LYS133, LYS133, ARG201, LYS222, HIS310, HIS310, GLU179, ASP307, GLU344, GHLU179, ALA200, ARG201, LYS 222, and HIS310 by forming five hydrogen bonds, seven hydrogens with electrostatic, and 23 electrostatic interactions (Fig 6E).

### 3.13 ADMET profiling

The pkCSM and SwissADME tools were used to perform the ADMET profiling for the chosen therapeutic agents. The ADMET profile helped to predict the drug's characteristics, such as absorption, distribution, metabolism, excretion, and most crucially, toxicity it may potentially cause within the human body.

DB03332 adhered to the Lipinski rule and had a bioavailability score of 0.55. The compound did not inhibit any of the CYP1A2, CYP2C19, CYP2C9, CYP2D6 AND CYP3A4 enzymes. Additionally, the medication DB07452 has a bioavailability score of 0.55 and adheres to the Lipinski rule. The compound does not inhibit the CYP1A2, CYP2C19, CYP2C9, CYP2D6, enzymes neither is a substrate of CYP3A4. The table provided below shows further pharmacokinetic characteristics of the medication. The medicinal molecule DB01262 does not adhere to one of the Lipinski rules with WLOGP > -0.4, as shown in Table 3. The medicinal molecule has a very low BBB permeability. In terms of its metabolism, the compound does not inhibit any of the CYP1A2, CYP2C19, CYP2D6, CYP2C9 enzymes and is not a CYP3A4 substrate. Furthermore, the medication also has a bioavailability score of 0.55. The DB02076 medication also violates 1 Lipinski rule; with NhorOH > 5 and has the lowest bioavailability scores of all the compounds of 0.11. Additionally, the compound does not exhibit BBB permeability and it does not inhibit any of the CYP2D6, CYP3A4, CYP2C9, CYP1A2 and CYP2C19 enzymes. Additionally, it was discovered that the pharmacological compound DB00727 drug's bioavailability score is 0.55 and thus does not adhere to the first Lipinski criterion. The compound does not show BBB permeability. Furthermore, the compound does not inhibit any of the CYP2D6, CYP1A2, CYP2C19 and CYP2C9 enzymes and does not function as a substrate for CYP3A4.

**Table 3:** The shortlisted five compounds’ ADME profiles measured against phospho-2-dehydro-3-deoxyheptonate aldolase.

DRUGBANK ID	DB03332	DB07452	DB01262	DB02076	DB00727
STRUCTURE	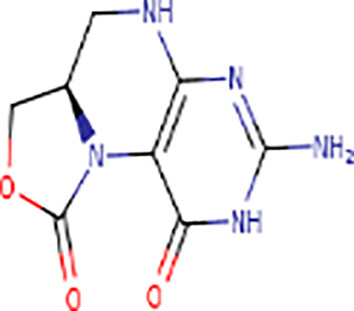	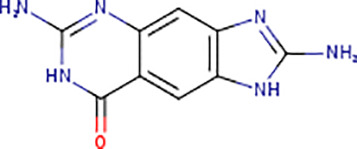	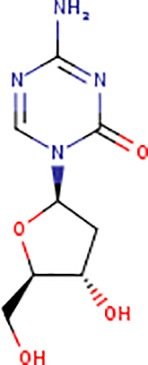	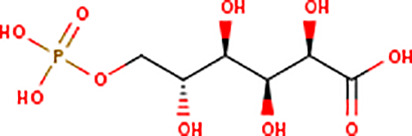	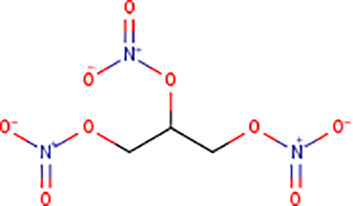
**DRUGLIKENESS**					
LIPINSKI	Yes; 0 violation	Yes; 0 violation	Yes; 0 violation	Yes; 1 violation: NHorOH > 5	Yes; 1 violation: NorO > 10
BIOAVAILABILITY SCORE	0.55	0.55	0.55	0.11	0.55
**ABSORPTION**					
Intestinal absorption (human)	68.644	68.886	62.235	0	73.177
Skin Permeability	-2.841	-2.735	-2.801	-2.735	-2.52
DISTRIBUTION					
BBB permeability	-0.781	-1.16	-1.091	-2.015	-1.706
CNS permeability	-3.31	-3.926	-3.647	-5.783	-3.278
METABOLISM					
CYP1A2 inhibitor	NO	YES	NO	NO	NO
Inhibitors of CYP2C19, CYP2C9, CYP2D6, CYP3A4, CYP2D6, and CYP3A4 substrate	NO				
**EXCRETION**					
Total Clearance	-0.078	1.05	0.555	0.527	1.084
Renal OCT2 substrate	NO	YES	NO	NO	NO

The toxicity characteristics of the five compounds have also been determined. As can be seen in Table 4, none of the five medications produced any PAINS alarms. In addition, none of the medicines exhibited AMES toxicity or skin sensitization. For DB03332, DB07452, and DB01262, the *T. pyriformis* toxicity is 0.228, 0.285 for DB07452, and 0.274 for DB01262, respectively. Furthermore, the toxicity table provided information on the medications’ hepatotoxicity, hERG I and hERG II inhibitor capacity, as well as the maximum tolerated dose in humans.

**Table 4:** The toxicity profiling for shortlisted five compounds against the phospho-2-dehydro-3-deoxyheptonate aldolase protein.

TOXICITY					
DRUGBANK ID	DB03332	DB07452	DB01262	DB02076	DB00727
AMES toxicity	NO	YES	NO	NO	YES
Max. tolerated dose (human)	0.661	0.424	1.236	0.84	0.445
hERG I inhibitor	NO	NO	NO	NO	NO
hERG II inhibitor	NO	NO	NO	NO	NO
Hepatotoxicity	YES	YES	YES	NO	NO
Skin Sensitization	NO	NO	NO	NO	NO
*Pyriformis* toxicity	0.228	0.285	0.274	0.285	0.232

## 4. Discussion

*Gardnerella vaginalis* is a species of Gram-variable-staining, facultative anaerobic bacteria. The organism is known for its small size, typically measuring between 1.0 and 1.5 μm in diameter, and it is non-spore-forming and non-motile. Morphologically, it is characterized by its coccobacilli shape. *G. vaginalis* is associated with bacterial vaginosis, a condition that can be either asymptomatic or accompanied by symptoms such as vaginal discharge, vaginal discomfort, and a malodorous, “fish-like” odor [39]. The amine whiff test is a diagnostic tool for bacterial vaginosis, which involves adding 10% potassium hydroxide (KOH) to the vaginal discharge to detect the presence of amines.

A positive outcome is determined by the presence of a distinct fishy odor. These tests can differentiate between vaginal symptoms produced by *G. vaginalis* and those caused by other species, such as *Trichomonas* and *Candida albicans*. The symptoms may be similar but they may need different therapies. *Trichomonas vaginalis* and *G. vaginalis* exhibit comparable clinical manifestations, including the production of a frothy gray or yellow-green vaginal discharge, pruritus, and a positive result on the “whiff-test.” To differentiate between the two, a wet-mount slide may be used. This involves diluting a sample of the vaginal epithelium and placing it on a slide for examination under a microscope. When seen under a microscope, Gardnerella bacteria may be identified by the presence of “clue cells,” which are squamous epithelial cells covered with bacteria [40]. *G. vaginalis* produces a pore-forming toxin, vaginolysin, which affects only human cells [41]. Protease and sialidase enzyme activities frequently accompany *G. vaginalis* [42–45].

The bacteria's classification as a member of the Haemophiles genus is based on its physical characteristics and preferred environment. However, the connection between this organism and vaginitis is still not fully understood [46]. In 1955, Gardner and Dukes provided a description of the microorganism in connection to BV [47]. Through five decades of comprehensive investigation, several risk factors linked to the development of BV have been identified. Nevertheless, the precise cause of this illness is still unknown because of its intricate nature, along with the absence of a dependable animal model [47]. Gardnerella may be advantageous in small amounts: It aids in maintaining a balanced pH level, which is the measurement of acidity against alkalinity that prevents harmful germs from entering your vaginal canal.

Therefore, to find a therapeutic target for *Gardenerella Vaginalis*, the subtractive genomics technique has been applied in this study. Retrieving the pathogen proteome, removing paralogous sequences, identifying sequences non-homologous to the human proteome, screening for essential genes using the Database of Essential Genes (DEG), identifying drug targets for the DrugBank database, and identifying virulence and resistant proteins are all examples of subtractive genomics applications [48]. As a result, the *Gardenerella Vaginalis* proteome consisting of 1139 proteins is eventually reduced to 10 proteins by the applied approach. In addition, three cytoplasmic proteins were prioritized on the basis of subcellular localization. Given its strategic location and crucial biological role, the phospho-2-dehydro-3-deoxyheptonate aldolase enzyme was chosen as a therapeutic target for treating Gardnerella vaginalis infections. This enzyme demonstrated the highest potential among the non-homologous proteins identified, making it a favorable candidate for drug targeting. Its importance in the metabolic pathways of the bacterium suggests that inhibiting this enzyme could disrupt the pathogen's survival and propagation. The other choices, however, might also be thought of as prospective targets for the development of medications using this technique [49]. Phospho-2-dehydro-3-deoxyheptonate aldolase is also known as 3-deoxy-D-arabino-heptulosonate 7-phosphate synthase DAHP synthase and Phospho-2-keto-3-deoxyheptonate aldolase [50]. Many organisms, including bacteria, plants, and fungi use the enzyme phospho-2-dehydro-3-deoxyheptonate aldolase (PDA) to create aromatic amino acids including phenylalanine, tyrosine, and tryptophan. It causes the condensation of 3-deoxy-D-arabino-heptulosonate 7-phosphate (DAHP) to produce phosphoenolpyruvate (PEP). Additionally, using the Modeller, the homology modelling technique was used to generate the protein structure [51]. After the validation process, molecular docking was conducted to assess the binding affinity of potential compounds to the target protein structure. This method involves simulating the interaction between the protein and small molecules, allowing researchers to predict how well the compounds bind to the protein's active site. A high binding affinity suggests that the compounds have the potential to inhibit the target protein effectively, providing a basis for the development of new therapeutic agents against *Gardnerella vaginalis* [52]. The library of FDA-approved medications was then screened against the identified drug target. The most potent FDA-approved medication compounds were chosen based on the binding affinities of the protein and the drug compound. Drug Bank ID: DB03332 (5,6-Cyclic-Tetrahydropteridine), one of these drug compounds, is utilized for Nitric Oxide Synthase that produces nitric oxide (NO) which is implicated in vascular smooth muscle relaxation through a cGMP-mediated signal transduction pathway. NO mediates vascular endothelial growth factor (VEGF). This drug is in clinical trial [53]. The DrugBank ID: DB07452 (2,6-diamino-1,7-dihydro-8H-imidazo[4,5-g] quinazolin-8-one), that produces tgt exchanges the guanine residue with 7-aminomethyl-7-deazaguanine in tRNAs with GU(N) anticodons (tRNA-Asp, -Asn, -His and -Tyr). After this exchange, a cyclopentendiol moiety is attached to the 7-am. This drug is also is in clinical trial [54]. The compound DB01262 (Decitabine) is a chemotherapeutic pyrimidine nucleoside analogue used for the treatment of Myelodysplastic Syndromes (MDS) by inducing DNA hypomethylation and corresponding alterations in gene expression. Current diseases being studied include Solid Tumors, Thalassemia, Sickle Cell disease, Renal Cell Carcinoma, and T-Cell Lymphoblastic lymphoma [55]. The DrugBank ID DB02076 corresponds to 6-phospho-D-gluconic acid. Its general function is Phosphogluconate dehydrogenase (decarboxylating) activity, which involves catalyzing the oxidative decarboxylation of 6-phosphogluconate to ribulose 5 phosphate and CO [2], while simultaneously reducing NADP to NADPH [53]. The compound DB00727, also known as Nitroglycerin, is a type of nitrate vasodilator. It is used to treat or prevent conditions such as angina, heart failure, hypertension, and anal fissures. Its main function is to act as a protein kinase. Specifically, it acts as a receptor for the atrial natriuretic peptide NPPA/ANP and the brain natriuretic peptide NPPB/BNP. These hormones are powerful vasoactive substances that play a crucial role in maintaining cardiovascular balance [56]. As a result, the findings of this investigation are presented below along with a recommendation that additional experimental research be conducted to get an insight into the impact the drug may have on our potential drug target.

## 5. Conclusion

The study's findings have paved the way for novel treatment strategies for infections caused by *Gardnerella vaginalis* by identifying a protein that is both druggable and essential to the bacterium's survival. Moving forward, a combinatorial approach that integrates *in silico* methods with other experimental techniques could enhance the discovery of more effective therapeutic strategies for treating *G. vaginalis* infections. This multidisciplinary approach may yield new avenues for drug development and improve patient outcomes.
